# Supplementary material for: Comprehensive Annotation and Functional Exploration of MicroRNAs in Lettuce
Source: Front Plant Sci. 2021 Dec 24;12:781836. doi: 10.3389/fpls.2021.781836 (PMC8739914; doi:10.3389/fpls.2021.781836)
Supplement: Supplementary file 2 [file Data_Sheet_8.ZIP › GSM2230747.plot/Lsa-miRN46_Lsat_1_v5_gn_8_63401.1_1397_TPlot.pdf]

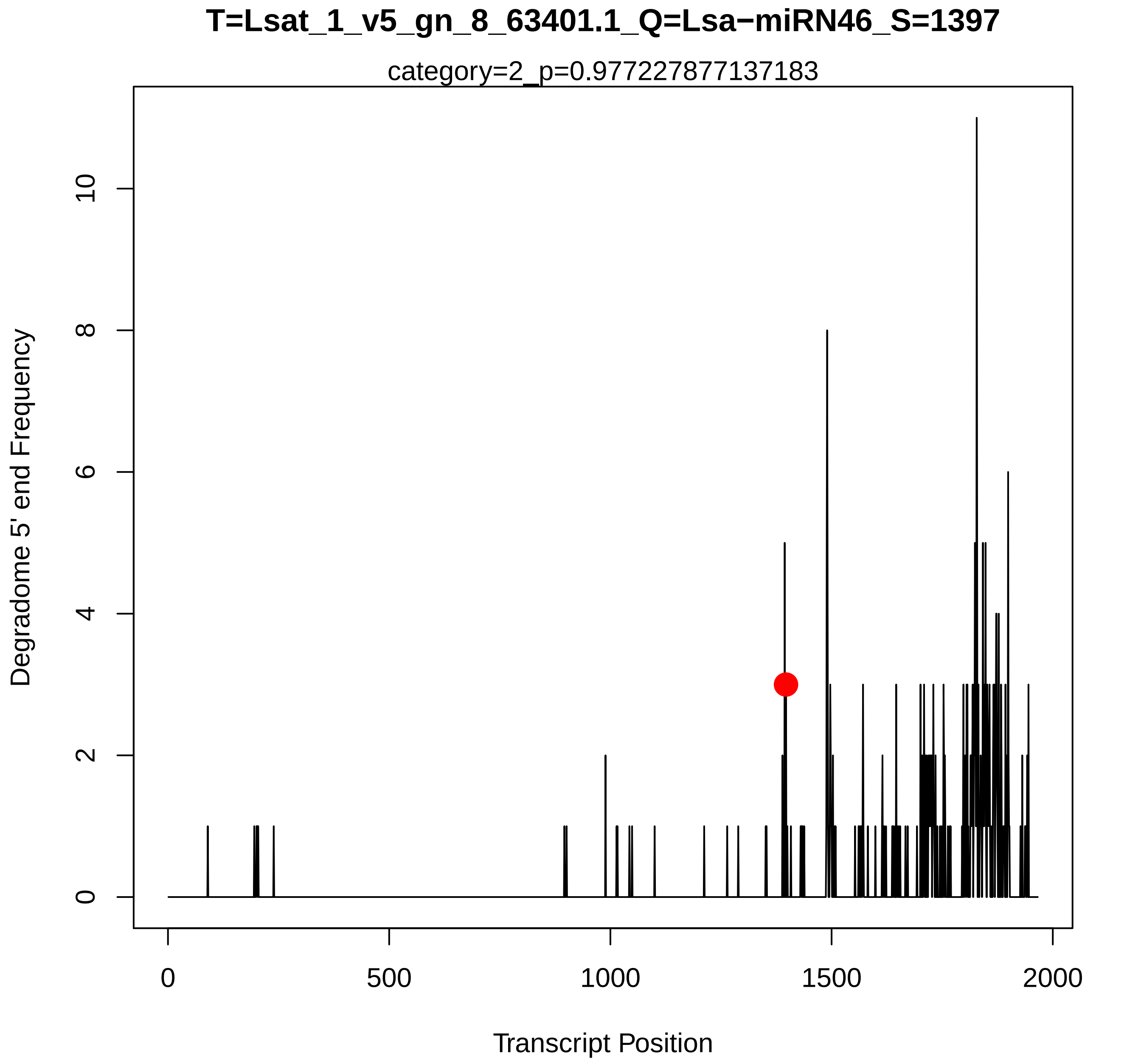
T=Lsat_1_v5_gn_8_63401.1_Q=Lsa−miRN46_S=1397
category=2_p=0.977227877137183
Degradome 5' end Frequency
0
2
4
6
8
10
Transcript Position
0
500
1000
1500
2000
